# Hospitalization in adults aged 75 years and older with type 2 diabetes: the role of kidney impairment and glycemic control

**DOI:** 10.1186/s12877-026-08386-7

**Published:** 2026-10-05

**Authors:** Bertin Magamba, Junmei Miao Jonasson, Louise Pettersson, Anders Halling, Björn Agvall

**Affiliations:** 1 Vårdcentralen Falkenberg, Region Halland, Sweden; 2 School of Public Health and community medicine, institute of medicine, Sahlgrenska academy, university of Gothenburg, Gothenburg, Sweden; 3 Research and Innovation Center, Region Halland, Halmstad, Sweden; 4 Department of Clinical Sciences, Division of Pathology, Lund University, Lund, Sweden; 5 Center for Primary Health Care Research, Department of Clinical Sciences, Lund University, Malmö, Sweden

**Keywords:** Type 2 diabetes, Aged, Chronic kidney disease, Glycosylated hemoglobin A, Hospitalization, Primary care

## Abstract

**Background:**

Older adults aged ≥ 75 years with type 2 diabetes (T2D) experience high healthcare utilization, yet evidence on treatment patterns and outcomes in this age group is limited.

**Objective:**

To assess associations between glycemic control, kidney function, and hospitalization among individuals aged ≥ 75 years with T2D.

**Methods:**

Individuals aged ≥ 75 years with T2D were identified in 2018 using data from a population‑based healthcare database covering all levels of care in Region Halland. Healthcare utilization, including hospital admissions, hospital days, primary care encounters and mortality, was evaluated during 2019. Inclusion required at least one measurement of glycated hemoglobin (HbA1c) and estimated glomerular filtration rate (eGFR). Kidney function was classified by eGFR ≥ 60; 30–59; <30 mL/min/1.73 m², and HbA1c as < 52, 52–70, or > 70 mmol/mol. Associations with hospital days were analyzed using Poisson regression adjusted for demographic, clinical, and treatment variables.

**Results:**

The cohort comprised 1,446 individuals (49% women); 88 patients (6%) died during follow‑up. HbA1c was < 52 mmol/mol in 43%, 52–70 mmol/mol in 45%, and > 70 mmol/mol in 12%. CKD was common, with 56% classified as stage 3 and 10% as stage 4–5. Hospital days were higher among individuals with CKD stage 4–5 (RR 1.93, 95% CI:1.71–2.17) and HbA1c > 70 mmol/mol (RR 1.44, 95% CI:1.28–1.61). Primary care encounters were frequent (physician visits: 4.6 [SD 3.7]; nurse visits: 9.0 [SD 9.3]) and associated with CKD but not with glucose dysregulation. Insulin use was associated with more hospital days, whereas metformin and incretin‑based therapy were associated with fewer.

**Conclusions:**

In adults aged ≥ 75 years with T2D, hospitalization burden was substantial and was associated with advanced CKD and poor glycemic control, while primary care encounters were frequent. These findings demonstrate an association between kidney function, glycemic status, and healthcare utilization in older adults with T2D.

**Supplementary information:**

The online version contains supplementary material available at https://doi.org/10.1186/s12877-026-08386-7.

## Key points

In individuals aged ≥75 years with type 2 diabetes, hospitalization was driven primarily by advanced chronic kidney disease and multimorbidity rather than glycemic control alone.

Higher HbA1c levels were independently associated with more hospital days, but with smaller effects than severe renal disease.

Older individuals with type 2 diabetes required both frequent primary care contacts and substantial hospital care, highlighting the complexity of multimorbidity in advanced age.

Insulin treatment was associated with increased hospital days in this older cohort, whereas metformin and incretin-based therapies were associated with fewer hospital days.

**Supplementary information:**

The online version contains supplementary material available at https://doi.org/10.1186/s12877-026-08386-7.

## Introduction

Type 2 diabetes (T2D) is a major public health challenge in Nordic countries, with prevalence rising steadily over recent decades due to lifestyle factors, environmental influences, and population ageing [1–3]. In Sweden, the proportion of individuals treated with glucose-lowering medications increased by more than 60% between 2006 and 2013, and similar trends have been reported in Norway and Denmark [1, 4, 5]. Older adults, particularly those aged 75 years and older, represent a vulnerable subgroup characterized by multimorbidity, frailty, and frequent exclusion from randomized clinical trials [6, 7]. This underrepresentation creates a significant evidence gap regarding the safety and effectiveness of diabetes treatments in this population [8].

T2D in older adults is associated with a high burden of complications, including cardiovascular disease and chronic kidney disease (CKD), which substantially influence prognosis and healthcare utilization [9, 10]. Impaired renal function is common in this age group and affects treatment choices, dosing, and risk of adverse events [11, 12]. Poor glycemic control and declining kidney function are linked to increased hospitalization and mortality [13–15]. While novel therapies such as sodium–glucose cotransporter 2 (SGLT2) inhibitors and glucagon-like peptide-1 (GLP-1) receptor agonists have demonstrated benefits for both glycemic control and renal outcomes [16, 17], their real-world use and impact among the oldest patients remain poorly understood.

In Sweden, the management of T2D occurs predominantly in primary care, where continuity and preventive strategies are essential [18, 19]. However, older patients with complex comorbidities often require frequent transitions between care levels, leading to higher resource consumption and risk of fragmented care [20, 21]. Understanding treatment patterns, renal function trajectories, and their association with healthcare utilization in this population is therefore critical for optimizing care delivery and resource allocation.

The objective for present study was to examine the association between glycemic control, kidney function, and treatment patterns with healthcare utilization among individuals aged 75 years or older with T2D in a population-based setting.

## Methods

### Study design and setting

This study was a retrospective, population-based cohort analysis using real-world data from Region Halland during year 2019, located in southwestern Sweden with approximately 340,000 inhabitants. The regional healthcare system provides both primary and secondary care and includes three acute hospitals, two emergency departments, and 48 primary care units. Data were obtained from the Regional Healthcare Information Platform (RHIP), which integrates clinical, demographic, and administrative information across all levels of care [22].

### Study population

The cohort comprised of individuals aged 75 years or older with a documented diagnosis of (ICD-10 codes E11 and E14). Patients were identified in 2018 as residents of Region Halland and included if they had at least one healthcare visit and registered measurements of glycated hemoglobin (HbA1c) and estimated glomerular filtration rate (eGFR) during 2019. The cohort was extracted based on 2018 residency and consisted of individuals who were alive at some point in 2019. The study period was the calendar year 2019. Patients younger than 75 years or without healthcare contact in the preceding year or during the study year were excluded.

### Data collection

Baseline demographic variables included age, sex. Comorbidities were identified using ICD-10 codes during a lookback period (2015–2019). The study identified T2D and relevant comorbid conditions using standardized ICD-10 codes. Hypertension was coded as I10. Atherosclerotic cardiovascular disease (ASCVD) included ischemic heart disease (I20–I29), peripheral arterial disease (I73.9), and heart failure (I50), while cerebrovascular disease was represented by I63–I69. Chronic obstructive pulmonary disease (COPD) was identified using code J44. Diabetic retinopathy was captured under codes H36 and E113. Clinical data comprised HbA1c, eGFR, systolic blood pressure urine- Albumin-to-Creatinine Ratio (UACR), and low-density lipoprotein (LDL) cholesterol. Laboratory data within RHIP are comprehensively collected across healthcare settings in Region Halland, enabling identification of individuals with renal impairment through recorded eGFR measurements and assessment of glycemic control through HbA1c measurements, irrespective of hospitalization status.

Treatment patterns were evaluated based on dispensed prescriptions of glucose-lowering and cardiovascular agents. Medication use was identified using ATC codes. Glucose-lowering therapies comprised insulin (A10A), metformin (A10BA), sulfonylureas (A10BB), DPP-4 inhibitors (A10BH), GLP-1 receptor agonists (A10BJ), and SGLT-2 inhibitors (A10BK). Cardiovascular therapies included beta-blockers (C07), renin–angiotensin system inhibitors (C09), calcium channel blockers (C08), statins (C10AA), ezetimibe (C10AX09), and diuretics (C03). During the study period, medication costs in Region Halland were managed through a centralized regional pharmaceutical budget rather than being allocated to individual healthcare centers. Local healthcare units had limited direct financial incentives affecting the selection of more costly glucose lowering therapies.

Healthcare utilization during the study period (January 1 to December 31, 2019) was assessed using data from the database RHIP. Measures included the number of contacts with primary care physicians and nurses, specialist visits, emergency department encounters, hospital admissions, and total inpatient care days. Hospitalizations were included only when the primary diagnosis was coded under ICD-10 I-codes (ASCVD) or E-codes (endocrinological conditions, primarily diabetes mellitus E11–E14); admissions outside these categories were excluded. Hospital outpatient care encompassed visits to physicians and nurses. Emergency department visits were classified as hospital outpatient care when they did not result in inpatient admission. Primary care visits to physicians and nurses were recorded separately and merged as primary care encounters. Individuals who died during 2019 were included, and their healthcare use was recorded up to the date of death. All-cause mortality data were obtained from RHIP.

### Statistical analysis

Descriptive statistics were reported as means with standard deviations for continuous variables and as frequencies and percentages for categorical variables. Group comparisons were performed using chi-2 tests for categorical variables. For continuous variables, one-way ANOVA was applied when comparing more than two groups, and the Mann–Whitney U test was used for non-normally distributed data when comparing two groups.

Glycemic control glycated hemoglobin (HbA1c) was categorized as good (< 52 mmol/mol), moderate (52–70 mmol/mol), or poor (> 70 mmol/mol). Kidney function was classified according to CKD stages based on eGFR [23, 24]. Stage 1–2 was defined as eGFR ≥ 60 mL/min/1.73 m². CKD stage 3 was defined as eGFR 30–59 mL/min/1.73 m². CKD stage 4–5 was defined as eGFR < 30 mL/min/1.73 m². To explore potential differences in treatment allocation according to kidney function, the distribution of glucose-lowering therapies across CKD stages was compared using chi-square tests.

The primary outcomes were healthcare utilization measured as hospital days. Associations between treatment patterns, glycemic control, kidney function, and outcomes were examined using Poisson regression for hospitalization days. The model was adjusted for age, sex, comorbidities, HbA1c, eGFR, and treatment variables. Poisson regression was considered appropriate. Overdispersion was assessed using the residual deviance degrees of freedom ratio and the negative binomial dispersion parameter. No significant overdispersion was detected and the dispersion parameter was close to zero.

Despite the availability of mortality data, the relatively low number of deaths during the study period (88 individuals, 6%) limited statistical power for multivariable survival analyses. Mortality was therefore described descriptively only, without adjusted regression modelling.

A two-sided p-value < 0.05 was considered statistically significant. Analyses were performed using IBM SPSS Statistics, version 31 (IBM Corp., Armonk, NY, USA).

## Results

A total of 1,446 individuals aged 75 years or older with T2D were included in the study. Of these, 605 (43%) had HbA1c < 52 mmol/mol, 650 (45%) had HbA1c between 52 and 70 mmol/mol, and 191 (12%) had HbA1c > 70 mmol/mol. The mean age was 79.5 years (SD 3.7) and was similar across HbA1c categories. Women accounted for 49% of the cohort. Hypertension was the most common comorbidity, present in 49% overall, followed by ASCVD (24%). Kidney function declined with higher HbA1c, with mean eGFR decreasing from 53.9 to 45.7 mL/min/1.73 m² across categories. CKD stage 3 was most frequent (56%), while advanced CKD (stage 4–5) was observed in 10% of participants, increasing from 6% in the lowest HbA1c group to 21% in the highest. The basic characteristics are presented in Table 1.

Dietary treatment was frequent at low HbA1c (24%) but rare at high levels (1%), while insulin use increased in frequency from 18% to 75% (*p* < 0.001). Metformin peaked in the intermediate group (65%) and declined among individuals with the highest HbA1c (51%). Incretin-based therapy increased from 14% to 51%, and SGLT-2 inhibitors from 2% to 12%. Cardiovascular medication was similar across groups except for diuretics, which increased from 35% to 48% (*p* = 0.004). Higher HbA1c was associated with more intensive glucose-lowering therapy, particularly insulin and incretin-based agents, while cardiovascular treatment patterns remained stable.

The distribution of glucose-lowering therapies differed significantly according to CKD stage which is seen Table 2. Insulin use was more common, whereas metformin and SGLT2 inhibitor use were less common, among individuals with advanced CKD.

Hospitalization burden increased with higher HbA1c and advanced CKD. Mean inpatient days rose from 1.5 in the lowest HbA1c group to 2.9 in the highest and from 1.6 in CKD stages 1–2 to 4.6 in stages 4–5 (both *p* < 0.001). Hospital admissions and ED visits showed similar gradients. Outpatient and primary care visits were relatively stable across HbA1c but increased with CKD severity, particularly nurse visits (13.3 in stage 4–5 vs. 7.1 in stage 1–2). The healthcare utilization is presented in Table 3.

Poisson regression analysis of hospital days adjusted for demographic, clinical, and treatment variables is presented in Table 3. Compared with CKD stages 1–2, stage 4–5 showed a higher risk (RR 1.93, 95% CI 1.71–2.17), and stage 3 indicated an increase (RR 1.15, 95% CI 1.05–1.25). Higher HbA1c categories were associated with greater hospital use (RR 1.52, 95% CI 1.39–1.65 for 52–70 mmol/mol and RR 1.44, 95% CI 1.28–1.61 for > 70 mmol/mol). ASCVD was associated with increased risk (RR 1.23, 95% CI 1.13–1.33). In the adjusted analysis, insulin use was associated with a higher number of hospital days (RR 1.18, 95% CI 1.08–1.29), while dietary treatment, metformin, and incretin-based therapy were associated with lower risk (RR 0.46, 95% CI 0.39–0.55; RR 0.62, 95% CI 0.56–0.68; and RR 0.76, 95% CI 0.69–0.82, respectively).

## Discussion

This population-based study provides a comprehensive description of healthcare utilization among adults aged 75 years and older with type 2 diabetes. Frequent encounters with primary care physicians and nurses coexisted with a substantial burden of hospital days. This pattern reflects a population characterized by frailty, reduced physiological reserve, and vulnerability to geriatric syndromes, factors likely influencing both outcomes and care needs. Kidney function was markedly impaired compared with younger populations. Advanced CKD and poor glycemic control measured during the study period were associated with greater hospitalization burden. Hospital use occurred despite intensive primary care follow up, alongside frequent use of DPP4 inhibitors, lower use of GLP1 receptor agonists, and widespread cardiovascular preventive therapy.

In this cohort of older adults aged 75 years and older with type 2 diabetes, the number of hospital days was substantially higher than previously reported in mixed-age or younger populations, indicating a pronounced absolute burden of healthcare utilization in this age group [15]. Both impaired kidney function and poor glycemic control were independently associated with increased hospitalization burden. Advanced chronic kidney disease demonstrated the strongest association, with individuals with CKD stage 4–5 experiencing nearly a two-fold higher rate of hospital days compared with those with preserved kidney function, whereas higher HbA1c levels were also associated with a significantly increased number of hospital days. The relative impact of advanced CKD on hospitalization was comparable to that reported in a population-based primary care study of younger individuals with T2D [15]. Consistent findings have also been reported in a population-based study of individuals with T2D and newly diagnosed cardiovascular disease, where impaired kidney function and poor glycemic control were strongly associated with hospitalization burden and adverse outcomes [19]. Together, these results confirm the importance of both renal function and glycemic regulation for healthcare utilization in older adults aged 75 years and older, while highlighting a substantially greater absolute burden of hospital days in this age group, likely reflecting multimorbidity and frailty. The pathophysiological link between chronic hyperglycemia and renal damage is well established, with prolonged exposure accelerating microvascular complications such as nephropathy and retinopathy [25, 26]. Declining estimated glomerular filtration rate further amplifies cardiovascular risk and mortality, as demonstrated in large registry studies [13, 27]. This pattern also reflects the complexity of managing multimorbidity in old adults and is consistent with previous findings that inadequate metabolic control and renal impairment drive higher rates of hospital admissions and emergency visits [10, 13].

Mortality during the one-year observation period was relatively low (6%), but deaths occurred more frequently among individuals with advanced CKD and higher HbA1c levels. The low absolute mortality was lower compared to other studies which is likely explained by the short follow-up period and the inclusion of a community-dwelling cohort with prevalent T2D rather than individuals selected at the time of acute cardiovascular events [15, 19, 28]. In contrast, longer-term population-based studies and cohorts enriched for cardiovascular disease have reported substantially higher mortality, typically ranging from 10 to 15% in primary-care populations and exceeding 20% in patients with newly diagnosed cardiovascular disease, with consistently higher risk observed in those with impaired kidney function and poor glycemic control [15, 19]. Thus, while absolute mortality differed, the direction and pattern of risk according to CKD severity and HbA1c were concordant across studies.

Treatment patterns in older adults with T2D reflect both therapeutic inertia and clinical caution. In present cohort, insulin use was frequent among individuals with poor glycemic control, while metformin and incretin-based therapies were associated with fewer hospital days, consistent with findings in broader populations [15]. Despite strong evidence supporting the cardio-renal benefits of SGLT2 inhibitors and GLP-1 receptor agonists, their use in individuals aged ≥ 75 years remained low in this population-based cohort [17, 18]. This is notable given the high prevalence of CKD and ASCVD in this age group and suggests that concerns related to age, frailty, renal function, and polypharmacy may outweigh potential benefits in routine practice. Treatment allocation varied considerably according to CKD stage (Supplementary Table 2). Individuals with advanced CKD were more frequently treated with insulin and less frequently treated with metformin or SGLT2 inhibitors. Therefore, the associations observed between glucose-lowering therapies and hospitalization burden are likely influenced by differences in kidney function, disease severity, and treatment selection, rather than reflecting direct effects of the therapies themselves. Insulin treatment was associated with a higher number of hospital days. This association likely reflects that insulin-treated individuals represent a subgroup with more advanced diabetes, greater comorbidity burden, and higher healthcare needs rather than a direct effect of insulin treatment itself. While statin therapy and newer glucose-lowering agents have been associated with reduced mortality in previous studies, the present analysis was not powered to formally evaluate treatment-related effects on mortality. Nevertheless, statin use was highly prevalent in this cohort, reflecting established evidence of cardiovascular benefit and reduced mortality risk in older adults reported in other populations [27]. Similarly, metformin and SGLT2 inhibitors were associated with reduced mortality in individuals with concomitant cardiovascular disease [19]. Patterns of therapeutic inertia were evident, with limited use of SGLT2 inhibitors and GLP-1 receptor agonists despite their proven cardio-renal benefits [16, 17]. Notably, the use of DPP4 was somewhat higher, indicating that caregivers prefer peroral pharmacotherapy rather than injection treating older individuals. These observations underline the persistent gap between guideline recommendations and real-world practice, particularly in old adults where concerns about frailty, polypharmacy, and adverse effects may influence prescribing decisions. The observed associations between glucose-lowering therapies and hospitalization burden should not be interpreted as causal effects of the therapies themselves. More likely, these findings reflect differences in disease severity, clinical complexity, and treatment selection rather than direct effects of the therapies themselves.

T2D is increasingly recognized as a heterogeneous disease entity, with distinct pathophysiological subgroups exhibiting different risks of complications, disease progression, and healthcare utilization [29]. Although classification according to recently proposed diabetes subgroups was not possible in the present study, such approaches may contribute to a more individualized understanding of hospitalization burden and outcomes in older adults with T2D. Future studies incorporating more detailed clinical and metabolic phenotyping may provide additional insights into the relationship between diabetes heterogeneity, CKD progression, and healthcare resource utilization.

A key finding of this study is the high level of engagement with primary care among older adults aged 75 years and older with type 2 diabetes, as reflected by frequent visits to both primary care physicians and nurses. This indicates that substantial efforts are made within primary care to support the ongoing management of this population. Frequent primary care contacts in this population may also reflect management of geriatric syndromes, including mobility limitations, polypharmacy, cognitive impairment, and need for care coordination, which extend beyond traditional disease specific management. Despite this intensive outpatient involvement, hospital admissions and the number of hospital days remained high, highlighting the complex healthcare needs associated with advanced age, multimorbidity, and progressive organ dysfunction. Importantly, the high hospitalization burden could be interpreted in the context of a relatively even higher level of primary care utilization, rather than as a lack of outpatient follow-up. As individuals age, increasing frailty and disease severity are likely to necessitate both intensified primary care contact and greater reliance on hospital care. Transitions between hospital and outpatient care nevertheless remain challenging, particularly following acute events [19, 30]. Evidence shows that structured follow-up and optimization of cardiometabolic therapy in primary care can reduce rehospitalization rates and improve clinical outcomes [31, 32], suggesting that continued efforts to strengthen coordination, continuity, and systematic post-discharge follow-up may further improve outcomes in this high-risk population, even in the context of already substantial primary care involvement.

### Strengths and limitations

A major strength of this study is the use of comprehensive, population-based data from both primary and hospital care, enabling a detailed assessment of real-world treatment patterns and outcomes. However, only individuals with documented eGFR and HbA1c measurements during the study period were included. This criterion possibly excluded severely ill patients for whom follow-up of T2D was not indicated or in focus due to other diseases. As a result, the mortality estimates in this analysis are probably slightly lower than could be expected. Furthermore, the objective was to investigate the association between glycemic control, kidney function, and healthcare outcomes in older adults with T2D, which guided the inclusion criteria and analytical approach. HbA1c and eGFR measurements were obtained during the same calendar year in which healthcare utilization was assessed. Consequently, temporal separation between clinical characteristics and outcomes was not possible, and some laboratory measurements may have been recorded after or during hospital admissions. Reverse temporal relationships therefore cannot be excluded. The findings should be interpreted as associations between clinical status and healthcare utilization during the study period rather than as evidence of predictive relationships. Due to the observational design, it was not possible to establish causality, and the findings represent associations. Additional limitations include the absence of data on frailty and functional status, which may influence both treatment decisions and outcomes.

Individuals without available HbA1c and eGFR measurements were excluded from the study. These patients may have differed systematically from those included, for example due to limited diabetes follow-up, frailty, competing comorbidities, or palliative care. Since healthcare utilization and mortality in the excluded population were not available, the representativeness of the study cohort relative to the entire population of older adults with T2D could not be fully assessed. The associations observed between glucose-lowering therapies and hospitalization burden should be interpreted with caution. Treatment selection is strongly influenced by clinical characteristics, including kidney function and disease severity. Consequently, the favorable associations observed for metformin and GLP-1 receptor agonists, and the adverse associations observed for insulin treatment, may partly reflect confounding by indication and differences in underlying disease severity rather than direct treatment effects.

Additional limitations include the absence of direct measures of frailty, functional status, and geriatric syndromes such as cognitive impairment and falls. These factors are highly prevalent in individuals aged 75 years and older and are likely to modify both treatment decisions and healthcare utilization, potentially leading to residual confounding.

### Conclusions

This study demonstrates that in adults aged 75 years and older with T2D, advanced CKD and suboptimal glycemic control are associated with a high hospitalization burden compared with younger populations. Renal function, glycemic status, and healthcare utilization were closely associated in this population, while evidence based cardiorenal therapies remained underutilized despite substantial need. These findings highlight the importance of management strategies that account for frailty, functional status, and multimorbidity, which are key drivers of vulnerability in this population. There is a clear need for age specific evidence to support individualized diabetes care aimed at reducing avoidable hospitalizations in the rapidly growing population of old adults.

## Supplementary information

Below is the link to the electronic supplementary material.

Supplementary Material 1

## Data Availability

The data that support the findings of this study are not publicly available due to privacy and ethical restrictions. Access to the data may be granted upon reasonable request to the corresponding author and subject to approval by the relevant ethical review authority and data holder.
