# Supplementary material for: Hospitalization in adults aged 75 years and older with type 2 diabetes: the role of kidney impairment and glycemic control
Source: BMC Geriatr. 2026 Oct 5;26:1254. doi: 10.1186/s12877-026-08386-7 (PMC13640178; doi:10.1186/s12877-026-08386-7)
Supplement: Supplementary file 1 [file 12877_2026_8386_MOESM1_ESM.docx]

**Table 1.** Baseline characteristics of the study cohort by HbA1c category.

|  | **Total** | **HbA1C <52 mmol/mol** | **HbA1c 52-70 mmol/mol** | **HbA1c >70 mmol/mol** | **p-value** |
| --- | --- | --- | --- | --- | --- |
| Total number for each group | 1446 | 605 (43) | 650 (45) | 191 (12) |  |
| Women | 710 (49) | 290 (48) | 318 (49) | 102(53) | 0.42 |
| Men | 736 (51) | 315 (52) | 332 (51) | 89 (47) |  |
| Age, mean (SD) | 79.5 (3.7) | 79.3 (3.5) | 79.6 (3.8) | 80.0 (3.8) | 0.053 |
| *Comorbidities* |  |  |  |  |  |
| Hypertension, n (%) | 706 (49) | 340 (56) | 293 (45) | 73 (38) | <0.001 |
| ASCVD, n (%) | 346 (24) | 175 (29) | 138 (21) | 33 (17) | <0.001 |
| Ischemic heart disease, n (%) | 245(17) | 120 (20) | 99 (15) | 27 (14) | 0.04 |
| Cerebrovascular insults, n (%) | 81 (6) | 47 (8) | 31 (5) | 3 (2) | 0.002 |
| Peripheral artery disease, n (%) | 11 (1) | 6 (1) | 5 (1) | 0 (0) | 0.39 |
| COPD, n (%) | 65 (4) | 32 (5) | 30 (5) | 3 (2) | 0.39 |
| *Kidney function* |  |  |  |  |  |
| eGFR ml/min, mean (SD) | 51.4 (15.5) | 53.9 (14.7) | 50.6 (15.6) | 45.7 (16.0) | <0.001 |
| CKD stage 1-2, n (%) | 477 (33) | 224 (37) | 212 (33) | 41 (22) | <0.001 |
| CKD stage 3, n (%) | 817 (56) | 342 (56) | 365 (56) | 110 (58) |  |
| CKD stage 4-5, n (%) | 152 (10) | 39 (6) | 73 (11) | 40 (21) |  |
| UACR, mean (SD) | 11.1 (31.6) | 8.4 (22.4) | 12.2 (37.2) | 15.0 (34.2) | 0.04 |
| UACR, n (%) <30 | 989 (68) | 413 (68) | 438 (67) | 138 (72) | 0.22 |
| UACR, n (%) 30-299 | 82 (8) | 27 (7) | 39 (9) | 16 (12) |  |
| UACR, n (%) >300 | 2(<1) | 0 (0) | 2(<1) | 0 (0) |  |
| Missing | 373 (26) | 165 (27) | 171 (26) | 37 (19) |  |
| LDL cholesterol, mean (SD) | 2.5 (1.0) | 2.5 (1.0) | 2.5 (1.0) | 2.5 (1.0) | 0.61 |
| LDL cholesterol >2.5, n (%) | 630 (13) | 270 (10) | 281 (14) | 94 (17) | 0.79 |
| Missing, n (%) | 816(44) | 335 (45) | 369 (43) | 97 (49) |  |
| *Blood pressure* |  |  |  |  |  |
| Systolic, mean (SD) | 138.4 (14.0) | 138.2 (13.8) | 138.5 (13.5) | 138.8 (16.0) | 0.85 |
| <130 mm Hg, n (%) | 341 (24) | 145 (25) | 151 (24) | 45 (24) | 0.99 |
| Deceased | 88 (6) | 23 (4) | 47 (7) | 18 (9) | 0.048 |
| *Diabetic pharmacotherapies* |  |  |  |  |  |
| Dietary (only), n (%) | 189 (13) | 143 (24) | 45 (7) | 1 (1) | <0.001 |
| Insulin, n (%) | 504 (35) | 112 (18) | 249 (38) | 143 (75) | <0.001 |
| Metformin, n (%) | 866 (60) | 348 (58) | 420 (65) | 98 (51) | 0.001 |
| Sulfonylurea, n (%) | 47 (3) | 10 (2) | 29 (4) | 8 (4) | 0.01 |
| Incretin‑based therapy, n (%) | 406 (28) | 87 (14) | 221 (34) | 98 (51) | <0.001 |
| DPP4, n (%) | 308 (21) | 71 (12) | 171 (26) | 66 (35) | <0.001 |
| GLP-1, n (%) | 128 (9) | 18 (3) | 68 (10) | 42 (22) | <0.001 |
| SGLT-2i, n (%) | 83 (6) | 10 (2) | 50 (8) | 22 (12) | <0.001 |
| *Cardiovascular pharmacotherapies* | |  |  |  |  |
| Betablockers, n (%) | 860 (60) | 345 (57) | 393 (60) | 122 (64) | 0.49 |
| RAASi | 964 (67) | 413 (68) | 431 (66) | 120(63) | 0.37 |
| Calcium blockers, n (%) | 584 (40) | 241 (40) | 268 (41) | 75 (39) | 0.47 |
| Statin, n (%) | 961(66) | 393 (65) | 435 (67) | 133 (67) | 0.10 |
| Ezetimib, n (%) | 25 (2) | 14(2) | 11 (2) | 0 (0) | 0.10 |
| Diuretics, n (%) | 551 (38) | 210 (35) | 249 (38) | 92 (48) | 0.004 |

Notes: n = number; SD = standard deviation; HbA1c = glycated hemoglobin; ASCVD = atherosclerotic cardiovascular disease; COPD = chronic obstructive pulmonary disease; CKD = chronic kidney disease; UACR = urine albumin-to-creatinine ratio; eGFR = estimated glomerular filtration rate; LDL = low-density lipoprotein cholesterol; RAASi = renin–angiotensin system inhibitors; SGLT-2i = sodium–glucose cotransporter-2 inhibitors; GLP-1 = glucagon-like peptide-1 receptor agonists; DPP-4 = dipeptidyl peptidase-4 inhibitors.

**Table 2.** Health care utilization in 2019 by HbA1c category and CKD stage.

|  |  | **Glycemic measurements** | | |  | **Renal function/CKD stages** | | |  |
| --- | --- | --- | --- | --- | --- | --- | --- | --- | --- |
|  | **Total** | **HbA1c**  **<52**  **mmol/mol** | **HbA1c**  **52-70**  **mmol/mol** | **HbA1c**  **>70**  **mmol/mol** | **p-value** | **Stages**  **1-2** | **Stage**  **3** | **Stages 4-5** | **p-value** |
| **Hospital care** |  |  |  |  |  |  |  |  |  |
| *Inpatient care****,*** *mean (SD)* | | | | | | | | | |
| Inpatient care days | 2.1 (6.3) | 1.5 (4.3) | 2.5 (6.1) | 2.9 (6.3) | <0.001 | 1.6 (4.9) | 2.0 (5.4) | 4.6 (7.0) | <0.001 |
| Hospital admissions | 0.4 (0.7) | 0.3 (0.9) | 0.4 (0.9) | 0.5 (0.8) | <0.001 | 0.3 (0.6) | 0.4 (0.8) | 0.8 (1.1) | <0.001 |
| ED | 0.7 (1.2) | 0.6 (1.2) | 0.7 (1.1) | 1.0 (1.4) | 0.006 | 0.6 (1.1) | 0.7(1.2) | 1.3 (1.5) | <0.001 |
| *Hospital outpatient care visits, mean (SD)* | | | | | | | | | |
| Physician | 0.4 (1.2) | 0.4 (1.2) | 0.4 (1.1) | 0.5 (1.4) | 0.31 | 0.3 (1.0) | 0.4 (1.0) | 0.9 (1.8) | <0.001 |
| Nurse | 0.7 (6.0) | 0.9 (9.1) | 0.4 (1.2) | 0.5 (1.6) | 0.32 | 0.3 (1.1) | 0.4 (1.9) | 2.8 (18.0) | <0.001 |
| **Primary care** |  |  |  |  |  |  |  |  |  |
| *Primary care visits, mean (SD)* | | | | | | | | | |
| Physicians | 4.6 (3.7) | 4.6 (3.7) | 4.6 (3.7) | 4.9 (4.0) | 0.60 | 4.2 (3.4) | 4.7 (3.6) | 5.5 (4.7) | <0.001 |
| Nurses | 9.0 (9.3) | 8.3 (10.1) | 9.5 (11.4) | 9.5 (9.2) | 0.08 | 7.1 (7.2) | 9.3 (10.8) | 13.3 (16.1) | <0.001 |

Notes: n = number; SD = standard deviation; HbA1c = glycated hemoglobin; CKD = chronic kidney disease; ED = emergency department.

Analyzed using one-way ANOVA for continuous variables.

**Table 3.** Poisson regression analysis of hospital days adjusted for demographic, clinical, and treatment variables.

|  | **RR** | **95% Wald CI for RR** | |  |
| --- | --- | --- | --- | --- |
|  |  | **Lower** | **Upper** | **p-value** |
| Age | 1.01 | 1.00 | 1.02 | 0.02 |
| Men | 1.04 | 0.97 | 1.12 | 0.25 |
| Women | 1.00 |  |  |  |
| ASCVD | 1.23 | 1.13 | 1.33 | <0.001 |
| HbA1c <52 | 1.00 | . | . | . |
| HbA1c 52-70 | 1.52 | 1.39 | 1.65 | <0.001 |
| HbA1c >70 | 1.44 | 1.28 | 1.61 | <0.001 |
| CKD stage 1-2 | 1.00 | . | . | . |
| CKD stage 3 | 1.15 | 1.05 | 1.25 | 0.003 |
| CKD stage 4-5 | 1.93 | 1.71 | 2.17 | <0.001 |
| Systolic bp <130 mm Hg | 0.95 | 0.88 | 1.04 | 0.25 |
| Dietary treatment | 0.46 | 0.39 | 0.55 | <0.001 |
| Insulin use | 1.18 | 1.08 | 1.29 | <0.001 |
| Metformin use | 0.62 | 0.56 | 0.68 | <0.001 |
| Incretin-based therapy use | 0.76 | 0.69 | 0.82 | <0.001 |
| Statin use | 1.05 | 0.97 | 1.13 | 0.27 |

Notes: RR = relative risk; CI = confidence interval; ASCVD = atherosclerotic cardiovascular disease; COPD = chronic obstructive pulmonary disease; HbA1c = glycated hemoglobin; CKD = chronic kidney disease; bp = blood pressure; Incretin-based therapy includes DPP-4 inhibitors and/or GLP-1 receptor agonists.
